# Influenza B/Victoria outbreak in a remote mountainous village: Oddar Meanchey Province, Cambodia, July–August 2023

**DOI:** 10.5365/wpsar.2025.16.3.1198

**Published:** 2025-08-20

**Authors:** Khemrin Pong, Sophanith Ung, Sengdoeurn Yi, Sovannara Long, Siphai Chhoung, Kimthy Nhim, Hayputhik Long

**Affiliations:** a Disease Prevention and Control Office, Kandal Provincial Health Department, Kandal, Cambodia.; b South Asia Field Epidemiology and Technology Network (SAFETYNET), Svay Rieng, Cambodia.; c Communicable Disease Control Department, Ministry of Health, Phnom Penh, Cambodia.; d Disease Prevention and Control Office, Oddar Meanchey Provincial Health Department, Oddar Meanchey, Cambodia.; e Disease Prevention and Control Office, Battambang Provincial Health Department, Battambang, Cambodia.

## Abstract

**Objective:**

A response team was deployed to rural Oddar Meanchey Province, Cambodia, in mid-August 2023, immediately after a cluster of patients with acute febrile illness was reported. The team aimed to identify the cause of the outbreak and analyse the epidemiological characteristics and associated risk factors.

**Methods:**

This retrospective cohort study involved all residents of Prasat Rumdoul Village. A case was defined as a resident with a fever ≥ 38 °C or a history of fever with symptoms such as cough, sore throat or coryza occurring from 18 July to  18 August. Demographic data, information about infection prevention practices and clinical information were collected using structured questionnaires and analysed using binomial regression. Laboratory samples were collected, and confirmatory laboratory tests and environmental investigations were also conducted.

**Results:**

Among the 126 villagers, 95 cases were identified (attack rate: 75.3%); 52 (54.7%) were female, and the median age was 29 years. Prolonged close contact with individuals who had influenza-like illness significantly increased the risk of infection (adjusted risk ratio [ARR]: 2.19, *P* = 0.002). Protective factors included mask-wearing (ARR: 0.26, *P* = 0.003) and regular handwashing (ARR: 0.85, *P* = 0.012). No villagers had been vaccinated against influenza within the past 1 year. Laboratory tests confirmed influenza type B/Victoria as the causative agent. Poor adherence to preventive measures and crowded living conditions contributed to the outbreak.

**Discussion:**

The outbreak was caused by influenza type B/Victoria, the same strain circulating in nearby Thailand. Public health interventions to improve vaccine accessibility and hygiene-promotion activities would be useful for preventing future outbreaks.

Influenza is a significant global health burden. The World Health Organization (WHO) has estimated that seasonal influenza causes up to 650 000 respiratory illness-associated deaths annually. (*1*) The disease leads to millions of severe cases worldwide, primarily affecting vulnerable populations such as children and older people, particularly during peak influenza season, which in Cambodia is from May to October. (*2*-*4*) The annual influenza-associated hospitalization rate for severe acute respiratory illness (SARI) in Cambodia is highest among children aged under 5 years, with a rate of up to 323 cases/100 000 population. (*5*) In 2016, the overall nationwide estimated burden of hospitalizations due to influenza-associated SARI was 7547 cases, causing substantial concern about the high rate of hospitalization among young children and older people. (*5*) A previous review of studies from 13 countries also found that older adults experienced a high rate of influenza-related morbidity. (*6*)

On 16 August 2023, the Oddar Meanchey Provincial Health Department was notified through the Cambodian Early Monitoring System of a cluster of 18 patients with acute febrile illness among 22 primary school students in Prasat Rumdoul Village, Ampil Commune, Oddar Meanchey Province. Initial hypotheses suggested dengue fever or chikungunya infection as the potential cause. The following day, a response team was deployed to confirm the diagnosis, describe the epidemiological characteristics of cases, identify factors associated with the illness and implement measures to control the outbreak; the team comprised staff from the Ministry of Health's Communicable Disease Control (CDC) Department, trainees from the field epidemiology training programme (FETP) and members of rapid response teams from the Provincial Health Department.

## Methods

### Epidemiological investigation

#### Study design and population

A retrospective cohort study was conducted to investigate the cluster of patients with acute febrile illness in Prasat Rumdoul Village. The study encompassed all village residents, including those who were ill and those who were not, to identify the cause of the outbreak and the factors associated with it.

#### Case definition

A case of influenza-like illness (ILI) was defined as a resident of Prasat Rumdoul Village who between 18 July and 18 August 2023 had a fever of ≥ 38 °C or a history of fever accompanied by at least one of the following symptoms: cough, sore throat or coryza.

#### Data collection

##### A paper-based structured questionnaire was used that had been designed by the investigation team before the interviews. The in-depth interviews were conducted during household visits by trained data collectors to gather information about sociodemographic characteristics, clinical signs and symptoms, preventive practices and exposure history. Due to the lack of access to formal medical records, all information was collected directly from participants through interviews.

The sociodemographic information collected included the respondent’s age, sex, education level and occupation. The clinical symptoms asked about included fever, cough, sore throat, runny nose and others. The use of preventive measures during the outbreak period was also assessed, such as the frequency of handwashing and mask-wearing practices. Routine hand hygiene practices evaluated included the use of soap and running water to wash hands, the use of alcohol-based hand sanitizers, or both. Participants were asked to report their handwashing frequency during the study period after contact with or exposure to objects or suspected ILI cases using four response options: “always,” “sometimes,” “rarely” or “never.” For analysis, these responses were coded into two categories: “always” and “sometimes” were coded as “yes,” while “rarely” and “never” were coded as “no.” Additionally, participants were asked to report how frequently they wore a mask during the study period when they went outside, met with people outside their household or were in crowded areas, with response options including “always,” “sometimes,” “rarely” or “never.” For analysis, these responses were coded into two categories: “always” and “sometimes” were coded as “yes,” and “rarely” and “never” were coded as “no.”

Respondents were also asked whether they had had close contact with individuals with ILI symptoms for a prolonged period. Close contact was defined as being within approximately 2 m of an individual with ILI symptoms for a prolonged period, such as sharing a living space, engaging in direct conversation or any other form of prolonged interaction. Participants were asked to report if they had any such close contact during this period, with response options being “yes” and “no.”

### Data analysis

Stata v. 17 (StataCorp, College Station, TX, USA) was used for data analysis. Descriptive statistics were used to summarize the demographic and behavioural characteristics; other factors were analysed as frequencies and percentages.

A binomial regression model was used to assess associations between risk factors and ILI. Variables with *P* < 0.20 were included in the multivariate binomial regression. *P* < 0.05 was considered statistically significant; the magnitude of the effect was assessed with the adjusted risk ratio (ARR) and 95% confidence intervals (CIs).

### Laboratory investigation

Blood samples were collected from symptomatic and recovered individuals to test for dengue, chikungunya and Zika virus infection. Nasopharyngeal samples from symptomatic individuals were collected and stored in a portable, insulated cooler before being sent to the National Institute of Public Health for reverse transcription–polymerase chain reaction (RT–PCR) testing to identify the influenza type and subtype. COVID-19 rapid diagnostic testing was also performed while the investigators were in the village.

### Environmental investigation

CDC Department staff and FETP trainees visited the village during the household survey to observe daily activities and assess adherence to public health and social measures during the outbreak period. Additionally, an informal meeting with village leaders was held to further understand community behaviours and the risk for communicable diseases.

## Results

### Epidemiological investigation

Among the 126 villagers, 95 ILI cases were identified (attack rate: 75.3%), 52 (54.7%) of whom were female; 31 (32.6%) cases had recovered before the investigation. The median age of cases was 29 years (range: 2–76 years). The investigation revealed varying incidence rates of ILI across demographic variables. The incidence among males was 70.5%, whereas among females it was 80.0% (**Table 1**). Among the different age groups, the highest incidences were observed in those aged 0–4 years and ≥ 50 years (80.0% each), followed by those aged 15–24 years (78.5%), those aged 5–14 years (77.1%) and those aged 25–49 years (71.1%) (**Table 1**). Attack rates by educational attainment ranged from 69.2% (for those who had completed secondary school to 80.0% (for those who had completed high school. Across occupations, service providers and children were the most affected groups. None of the people in the village had been vaccinated against influenza. Among the 95 ILI cases, 52 patients (54.7%) reported hospitalization at either a private clinic or public hospital (data not shown).

**Table 1:** Factors associated with an outbreak of influenza-like illness, Oddar Meanchey Province, Cambodia, 2023

Variable	No. villagers (*n* = 126)	No. ILI cases (% incidence) (*n* = 95)	Crude risk ratio	95% CI	*P*	Adjusted risk ratio	95% CI	*P*
**Sex**								
**Male**	**61**	**43 (70.5)**	**Reference**			**–**	**–**	**–**
**Female**	**65**	**52 (80.0)**	**1.13**	**0.92–1.39**	**0.221**	**–**	**–**	**–**
**Age group (years)**								
**0–4**	**10**	**8 (80.0)**	**Reference**			**–**	**–**	**–**
**5–14**	**35**	**27 (77.1)**	**0.96**	**0.67–1.38**	**0.842**	**–**	**–**	**–**
**15–24**	**14**	**11 (78.5)**	**0.98**	**0.64–1.48**	**0.932**	**–**	**–**	**–**
**25–49**	**52**	**37 (71.1)**	**0.88**	**0.62–1.26**	**0.518**	**–**	**–**	**–**
** ≥ 50**	**15**	**12 (80.0)**	**1**	**0.67–1.49**	**1.000**	**–**	**–**	**–**
**Education level**								
**No school**	**51**	**39 (76.4)**	**Reference**			**–**	**–**	**–**
**Primary school**	**57**	**43 (75.4)**	**0.98**	**0.79–1.21**	**0.900**	**–**	**–**	**–**
**Secondary school**	**13**	**9 (69.2)**	**0.90**	**0.61–1.34**	**0.620**	**–**	**–**	**–**
**High school**	**5**	**4 (80.0)**	**1.04**	**0.65–1.66**	**0.849**	**–**	**–**	**–**
**Occupation**								
**Farmer**	**70**	**50 (71.4)**	**Reference**			**–**	**–**	**–**
**Student**	**35**	**26 (74.2)**	**1.04**	**0.81–1.32**	**0.754**	**–**	**–**	**–**
**Child**	**13**	**11 (84.6)**	**1.18**	**0.89–1.55**	**0.227**	**–**	**–**	**–**
**Service provider^a^**	**8**	**8 (100.0)**						
**Travel during 14 days between 18 July and 18 August 2023**								
**Yes**	**21**	**18 (85.7)**	**1.16**	**0.94–1.44**	**0.244**	**–**	**–**	**–**
**No**	**105**	**77 (73.3)**	**Reference**					
**Influenza vaccination within the past 1 year**								
**Yes**	**0**	**0**	**–**	**–**	**–**	**–**	**–**	**–**
**No**	**126**	**95 (75.4)**	**–**	**–**	**–**	**–**	**–**	**–**
**Presence of chronic disease**								
**Yes**	**19**	**14 (73.6)**	**0.97**	**0.72–1.29**	**0.855**	**–**	**–**	**–**
**No**	**107**	**81 (75.7)**	**Reference**			**–**	**–**	**–**
**Close contact with ILI cases between 18 July and 18 August 2023**								
**Yes**	**92**	**86 (93.4)**	**3.53**	**2.01–6.19**	** < 0.001**	**2.19**	**1.34–3.57**	**0.002**
**No**	**34**	**9 (26.4)**	**Reference**					
**Regular mask-wearing behaviour**								
**Yes**	**26**	**4 (15.3)**	**0.16**	**0.06–0.41**	** < 0.001**	**0.26**	**0.11–0.63**	**0.003**
**No**	**100**	**91 (91.0)**	**Reference**					
**Hand hygiene behaviour (regular handwashing)**								
**Yes**	**46**	**22 (47.8)**	**0.52**	**0.38–0.71**	** < 0.001**	**0.85**	**0.76–0.96**	**0.012**
**No**	**80**	**73 (91.2)**	**Reference**					

CI: confidence interval; ILI: influenza-like illness; –: not applicable.

^a^ This category includes two elders, two motorcycle repairers, two sellers, one deputy village chief and one village chief.

**Fig. 1:**
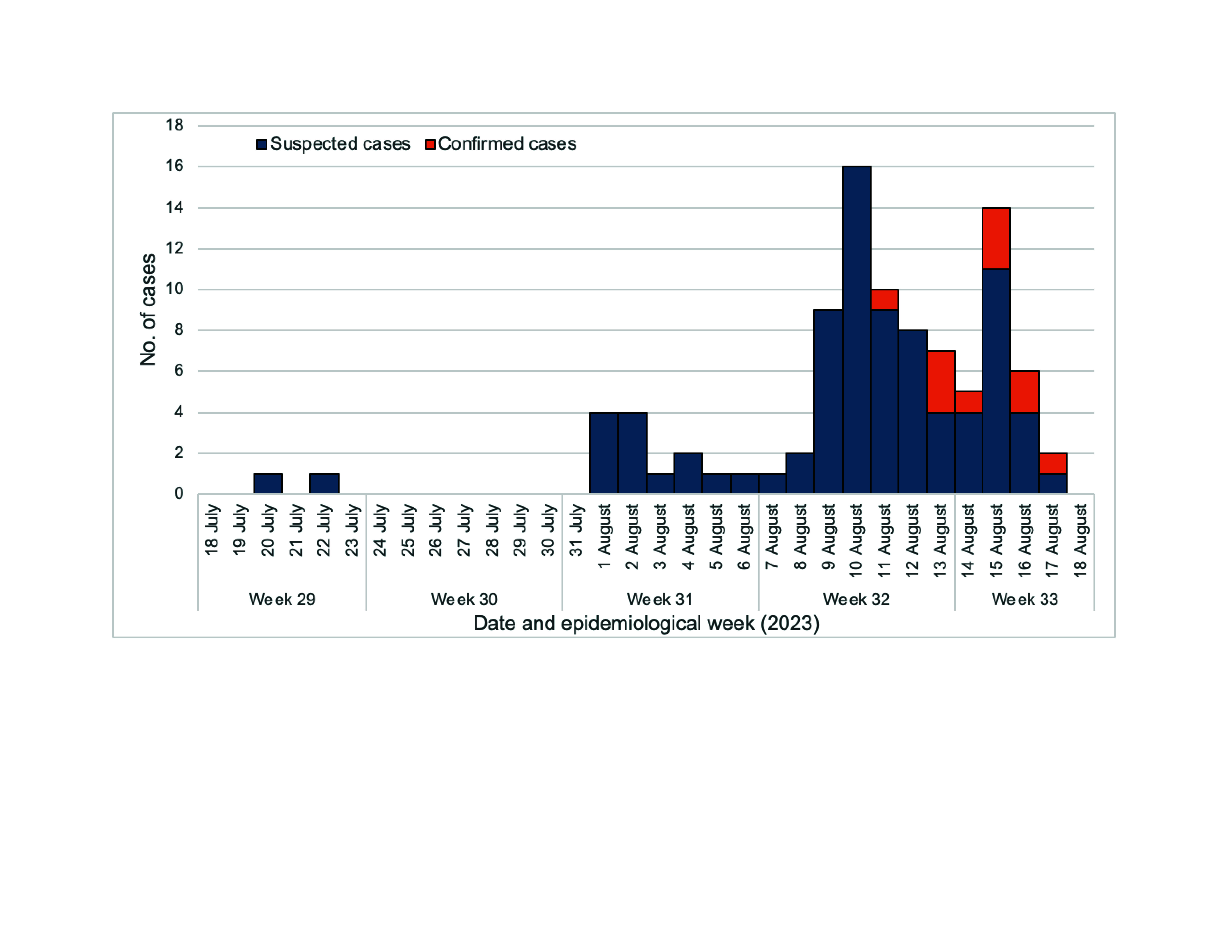
Epidemic curve of an outbreak of influenza-like illness in Prasat Rumdoul Village, Oddar Meanchey Province, Cambodia, 18 July to 18 August 2023 (n = 95)

The epidemic curve of the ILI outbreak was developed based on the household survey, covering cases occurring from 18 July to 18 August 2023. The suspected index case occurred on 20 July, and the highest number of cases recorded in a single day was 16 on 10 August. The daily number of cases then began a decreasing trend, with a secondary peak of 14 cases on 15 August (**Fig. 1**).

[**insert** Figure 1]

Regarding clinical signs and symptoms among patients, fever was the most common symptom (100%), followed by coughing or sneezing (83, 87.3%), sore throat (75, 78.9%), headache (73, 76.8%), myalgia (69, 72.6%) and runny nose (66, 69.4%). Orbital pain was reported by 58 (61.1%) patients, while fatigue affected 55 (57.9%). Less common symptoms included abdominal pain (35, 36.8%), vomiting (18, 18.9%), nausea (13, 13.7%) and rash (10, 10.5%).

Prolonged close contact with individuals with ILI between 18 July and 18 August significantly increased the risk of ILI (ARR: 2.19, 95% CI: 1.34–3.57, *P* = 0.002). However, those who reported regularly wearing a mask had a reduced likelihood of developing ILI (ARR: 0.26, 95% CI: 0.11–0.63, *P* < 0.003), as did individuals who practised regular handwashing (ARR: 0.85, 95% CI: 0.76–0.96, *P* = 0.012). Age group, education level, occupation, travel outside of the village for 14 days between 18 July and 18 August, influenza vaccination history and the presence of chronic illness were not associated with infection.

### Laboratory investigation

The 11 nasopharyngeal samples collected from cases who were ill at the time of collection all tested negative for COVID-19 via both the rapid diagnostic test and RT–PCR; however, 10 of those 11 samples tested positive for influenza type B/Victoria (data not shown). Twenty-two blood samples drawn from suspected cases who were still presenting with signs and symptoms and from cases who had already recovered tested negative for dengue, chikungunya and Zika virus (data not shown). Blood samples could be collected only from this subset of ILI cases due to limited resources.

### Environmental investigation

The environmental investigation conducted by staff from the CDC Department and FETP trainees revealed several significant factors related to the outbreak in the village. The majority of residents exhibited poor adherence to influenza prevention measures, such as mask-wearing, handwashing and physical distancing. It is common practice for villagers to visit others when they are sick, contributing to the rapid spread of communicable diseases. Although the village is small, it is overcrowded, which further facilitates disease transmission, and its remote location makes it challenging to effectively implement and monitor public health interventions. Additionally, many villagers migrate to Thailand for work, thus posing the risk of introducing and spreading infectious diseases. The geographical isolation of the village, situated along the mountainous border with Thailand, impacts access to health-care services and public health resources, complicating efforts to manage and contain disease outbreaks.

### Prevention and control measures

Community- and school-based education was initiated immediately to increase awareness about influenza, focusing on prevention methods, recognizing signs and symptoms, and basic management practices, such as encouraging rest and hydration, isolating symptomatic individuals, and seeking care from health facilities if needed. In addition, surveillance was enhanced at the health centre near the village to closely monitor trends in influenza cases and ensure the timely detection of any new infections.

## Discussion

This influenza outbreak affected the majority of the village’s population. The outbreak is a lens into patterns of influenza epidemiology in a community through the clinical signs and symptoms of the illness, the sociodemographic characteristics of the people in the village, their preventive practices and laboratory results, and the environmental factors associated with the outbreak.

The clinical signs and symptoms reported by cases were compatible with influenza signs and symptoms described by WHO and UpToDate guidelines, (*7*, *8*) and the epidemic curve suggested a propagated (i.e. progressive source) epidemic, spread by person-to-person transmission, with an incubation period consistent with the spread of influenza (i.e. 2–7 days). The epidemiological findings were strongly supported by environmental findings and laboratory results, the latter of which confirmed infection with influenza type B/Victoria. During this period, sentinel site data in Cambodia for ILI and SARI indicated predominantly influenza A(H1N1)pdm and no influenza B/Victoria. From the same surveillance system, influenza B/Victoria and predominant influenza A(H1N1)pdm were observed to be circulating in neighbouring Thailand. (*9*)

This influenza outbreak affected slightly more females than males, while there was no statistically significant difference between age groups, regardless of a respondent’s education level or occupation. WHO recommends that children aged under 5 years, adults aged over 65 years and people with chronic illnesses receive an annual influenza vaccination. (*10*) However, no one in the village where the outbreak occurred had been vaccinated because the vaccine was not available locally.

A history of travelling outside of the village was not associated with being a case. However, having close contact with someone with ILI between 18 July and 18 August 2023 was associated with becoming a case, while good practices such as regular mask-wearing and good hand hygiene were protective factors. It is likely that the source of infection was within the community.

Having a chronic disease was not associated with becoming a case. However, as much as 55% of the cases were reported to have been hospitalized due to growing concern over dengue illness. In this outbreak, no deaths or severe cases were reported. A previous study indicated that seasonal influenza B has a wide spectrum of clinical presentations, from mild upper respiratory tract symptoms to death from respiratory failure, depending on characteristics such as older age or the presence of a pre-existing medical condition. (*11*)

### Limitations

The index case had no history of travel outside the village or contact with suspected ILI cases, so the original source of infection for the index case could not be identified. Recall bias may have been introduced into the study due to reliance on retrospective data collection through participant interviews about symptoms and preventive measures. In addition, the village is quite small, with a population of 126, which may reduce the statistical power of the study. An additional qualitative study assessing environmental and sociobehavioural practices may help solidify our findings.

### Conclusions

The outbreak was confirmed to have been caused by influenza type B/Victoria, which affected the majority of the community’s population, and the highest attack rates were among children aged 0–4 years and adults aged ≥ 50 years. Lack of access to vaccination and having close contact with ILI patients were contributors to the outbreak, while measures such as mask-wearing and good hand hygiene practices proved to be protective. It is essential to improve the availability and accessibility of influenza vaccine, especially for vulnerable populations. Increasing community awareness about influenza through public health campaigns that emphasize the importance of good hygiene practices, such as handwashing and mask-wearing, especially during outbreaks, would be useful for controlling future outbreaks.
